# The impact of hygienic living conditions on the differentiation of male body height at the beginning of the twentieth century in the USSR

**DOI:** 10.1186/s40101-024-00367-2

**Published:** 2024-08-27

**Authors:** Lidia Lebedeva, Elena Godina

**Affiliations:** 1 N. N. Miklukho-Maklai Institute of Ethnology and Anthropology, Russian Academy of Sciences, Lenin Ave., 32A, Moscow, 119334 Russia; 2 Anuchin Research Institute and Museum of Anthropology, Lomonosov Moscow State University, Mokhovaya St., 11, Moscow, 125009 Russia

**Keywords:** Body height, Men, Russia, Twentieth century, Living conditions, Level of morbidity

## Abstract

**Background:**

The positive changes in hygienic living conditions are commonly believed to explain secular changes in body height and the age of maturity. However, it is difficult to estimate the separate impacts of these factors due to the lack of social and economic data and variations in the sources of information.

We hypothesized that final male body height could be associated with various socioeconomic indicators, such as the development of the medical care system, the quality of nutrition, and the level of sanitary and hygienic conditions. Moreover, we hypothesized that male body height could be associated with the level of morbidity in the region during the time of conscript childhood (from 1 to 7 years old).

**Materials and methods:**

We used two main sources of information in the analyses. The first is the data from the Statistical Reference Book published by the Central Statistical Committee in 1929. The second is the annual data from the Statistical Reference Book published in the Russian Empire. Since the conscripts were born between 1906 and 1909, we used datasets from 1910 to 1913. To analyze the data, we used a method of analyzing interacting variables called St. Nicolas House Analysis (SNHA).

**Results:**

Our analyses revealed direct associations between the morbidity of some diseases and male body height and other anthropometric parameters.

**Conclusions:**

There are associations between conscript final body height and the morbidity of influenza, dysentery and some venereal diseases, such as chancroid and syphilis. There were no associations between conscript final body height and the level of morbidity during childhood. However, other final parameters, such as BMI, weight, and chest circumference, could be associated with the morbidity of malaria, scabies, scurvy, and scarlet fever during childhood. The prevalence of these diseases could be strongly connected with unfavorable living conditions. The results are similar for both urban and rural areas.

## Background

The most common explanations for secular changes in body height and age at maturity are improvements in quality of life, such as advances in hygiene, medical care, and nutrition, as well as decreases in morbidity and infant mortality [1–5]. However, the overall and separate impacts of these factors are difficult to estimate due to the lack of social and economic data and differences in sources of information. Moreover, administrative changes in the territory units sometimes made such studies impossible due to the relevant changes in the reference books.

In Russia, we have only a few reliable sources of information that could be considered for studying changes in body height in the nineteenth and twentieth centuries for the territory of the whole country [6–9]. None of those sources of information provided initial socioeconomic data about the various indicators of quality and level of life to consider in the analyses and to find a proper explanation of the changes in body height. By good fortune, we found the reference book, the Statistical Reference Book, which was published by the Central Statistical Committee in 1929 [10]. This reference book contains information about male body height that matches the values of male body height from the most reliable source of information (Bunak’s article, 1932) up to two points after the comma. The reliability of this source of information was proven by other studies on temporal and spatial secular changes in Russia and neighboring countries [11, 12]. Additionally, in the book, a variety of socioeconomic indicators were published that could be included in the analyses of factors impacting human growth and development.

Using this source of information in the analyses is an opportunity to highlight the possible reasons for secular changes in Russia that took place after the 1920s [11].

The aim of this study was to estimate the factors that could influence male body height in Russia in the 1920s. We hypothesized that final male body height could be associated with various socioeconomic indicators, such as the development of the medical care system, the quality of nutrition, and the level of sanitary and hygienic conditions. Moreover, we hypothesized that male body height could also be associated with the level of morbidity of some diseases during the time of conscript’s childhood (from 1 to 7 years old).

## Materials

We used two main sources of information in the analyses. The first is the data from the Statistical Reference Book published by the Central Statistical Committee in 1929 [10]. It was used to study the impact of various socioeconomic factors on male body height. From this dataset, we used data on the body height and weight of the conscripts. Twenty-nine territories and more than 20 socioeconomic indicators were considered in the analyses. We chose socioeconomic indicators that are important for evaluating quality of life. In Table 1, we provide the title of the indicators and factors that explain which aspects of life conditions could be accounted for.

**Table 1:** The indicators of quality and level of life in the USSR in the 1920s [10]

No	The title of the indicator	Year, that the indicator accounted for	Factors of quality of life
1.	Population density, pers. per sq. km.	1928	Impact of economic activity
2.	Migration activity (the difference between those who came to the region and left it)	1928	Impact of economic activity
3.	Infant mortality under 1 year of age (per 1000 births, died before 1 year of age)	1927	The development of the healthcare system
4–5.	Distribution of potatoes for personal consumption in the city/in the village, thousand tons	1926/1927	Quality of nutrition
6–7.	Distribution of meat and lard for personal consumption in the village/in the city, thousand tons	1926/1927	Quality of nutrition
8–9.	Male literacy in villages and towns, %	1926	Level of education
10.	The number of crimes committed against a person (murders, fights etc.)	1928	Security of the territory
11.	The number of crimes committed against property (robbery etc.)	1928	Security of the territory
12–24.	Morbidity of some illnesses: dysentery, diphtheria, pulmonary tuberculosis, influenza, syphilis, chancroid, gonorrhea, scurvy, trachoma, malaria, smallpox, scarlet fever, measles	1927	Level of sanitary and hygienic conditions

The second is the annual data from the Statistical Reference Book published in the Russian Empire. As the conscripts were born in 1906–1909, we used the datasets for the years from 1910 to 1913. These datasets were used to assess the impact of different illnesses on morbidity during the conscript’s childhood. We calculated the average morbidity rate for 4 years. Forty-four regions were included in the analyses [13] (Table 2).

**Table 2:** The title and type of the illnesses used in the analyses, 1910–1913, 1929 [9, 11]

The type of diseases	The Statistical Reference Books for Russian Empire, 1910–1913	The Statistical Reference Book for the USSR, 1929
Children’s	Diphtheria	Diphtheria
	Measles	Measles
	Whooping _cough	–
	Scarlet fever	Scarlet fever
Respiratory	Influenza	Influenza
	Pulmonary tuberculosis	Pulmonary tuberculosis
Social-hygienic	Scurvy	Scurvy
	Trachoma	Trachoma
	Scabies	–
Sexually transmitted	Chancroid	Chancroid
	Syphilis	Syphilis
Others	Malaria	Malaria

It is worth mentioning that in the analyses, we used the men’s body height for rural and urban areas according to the exact territory. We considered that the inner borders of the country changed greatly from 1913 to 1929 and removed those territories that had completely changed their borders from the dataset.

## Methods

Spearman correlation ratios and controlled *p* values were used to estimate groups of socioeconomic factors. St. Nicolas House Analysis (SNHA) was used for further analysis of the associations between interacting variables [14].

The key advantage of the SNHA package is that it explores interacting variables by searching association chains where correlation coefficients between variables drop in a regular order between a set of variables.

While more typical methods in this area are visualization of pairwise correlations, principal component analysis, or multidimensional scaling, the SNHA package provides an alternative approach by uncovering ordered sequences of correlation coefficients, which can also be reversed. Thus, the basic assumption of this method is that the correlation coefficients between two variables, where one variable directly influences the other, are larger than those of secondary associations. Therefore, if we suggest that a variable A influences a variable B and B influences C, it can be assumed that r(AB) > r(AC) and that in the opposite direction, r(CB) > r(CA). The existence of such association chains with the correct ordering of three or more nodes is much less likely to exist by accident than significant pairwise correlations. Therefore, the SNHA method has very limited requirements for choosing thresholds. Basically, they are the meanings of the *p* value and the correlation ratio [14–16].

The correlation chains, existing in the dataset, are translated into edges between the variables. The variables become nodes of a graph. The graph can then be visualized, and the major relations between the variables are visible.

Moreover, we can use bootstrapping to evaluate and illustrate the quality of the chains. The solid line between the nodes indicates that this edge (association) was found in more than 75% of all resamplings of our data. A broken line indicates that the edge was found in more than 50% of all resamplings, and a dotted line indicates that the edge was found in more than 25% of all resamplings [14, 16]. Also, the SNHA package allows the calculation of the R-squared values for a given dataset, graphs, and nodes based on the linear regression model. All figures in this manuscript and statistical analyses were performed using R version 4.3.1 (released on 16.06.2023) [17].

## Results

First, we estimated that the correlation ratios between various socioeconomic factors and body height in urban and rural areas were nonsignificant for all variables except for the level of morbidity of some diseases (Table 3).

**Table 3:** The correlation ratios between various socioeconomic factors and body height in urban and rural areas

No	The title of the indicator	Body height in urban areas, 1928		Body height in rural areas, 1928	
		*r*	*p* value	*r*	*p* value
1.	Population density, pers. per sq. km.	− 0.31	0.1048	− 0.30	0.1262
2.	Migration activity (the difference between those who came to the region and left it)	0.26	0.2029	0.26	0.2105
3.	Infant mortality under 1 year of age (per 1000 births, died before 1 year of age)	− 0.37	0.1784	− 0.32	0.2483
4.	Distribution of potatoes for personal consumption in the city, thousand tons	0.22	0.3325	0.16	0.4941
5.	Distribution of potatoes for personal consumption in the village, thousand tons	− 0.03	0.8953	− 0.08	0.7432
6.	Distribution of meat and lard for personal consumption in the city, thousand tons	0.13	0.5948	0.16	0.5038
7.	Distribution of meat and lard for personal consumption in the village, thousand tons	0.26	0.2726	0.26	0.2734
8.	Male literacy in towns, %	− 0.19	0.3344	− 0.15	0.4510
9.	Male literacy in villages, %	− 0.26	0.1780	− 0.28	0.1547
10.	The number of crimes committed against a person (murders, fights etc.)	− 0.18	0.3662	− 0.25	0.2164
11.	The number of crimes committed against property (robbery etc.)	− 0.20	0.3134	− 0.27	0.1654
12–24.	Morbidity of some diseases	See Figs. 1 and 2.

**Fig. 1:**
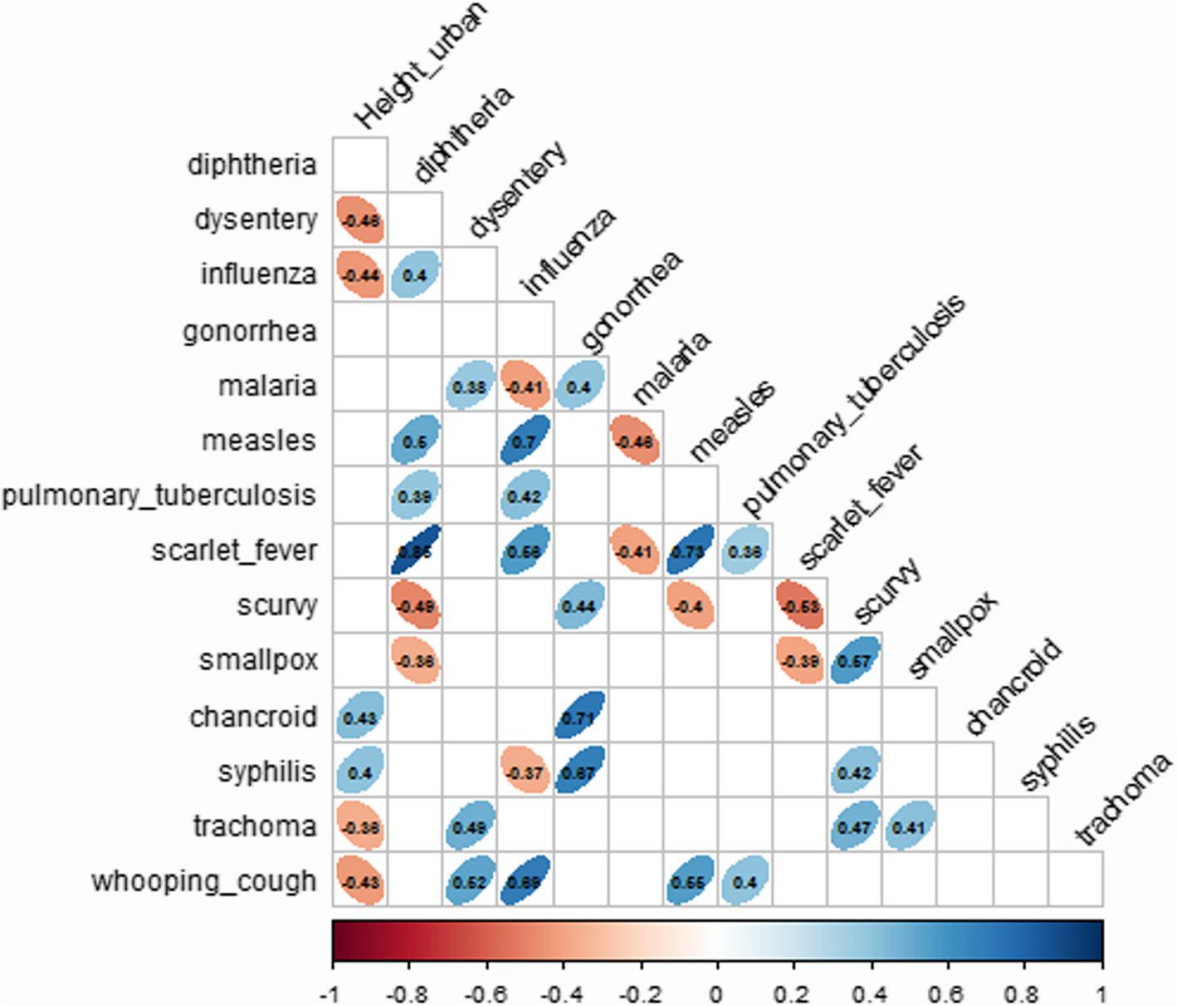
The correlation ratio: morbidity levels of certain illnesses vs. male body height in urban areas

**Fig. 2:**
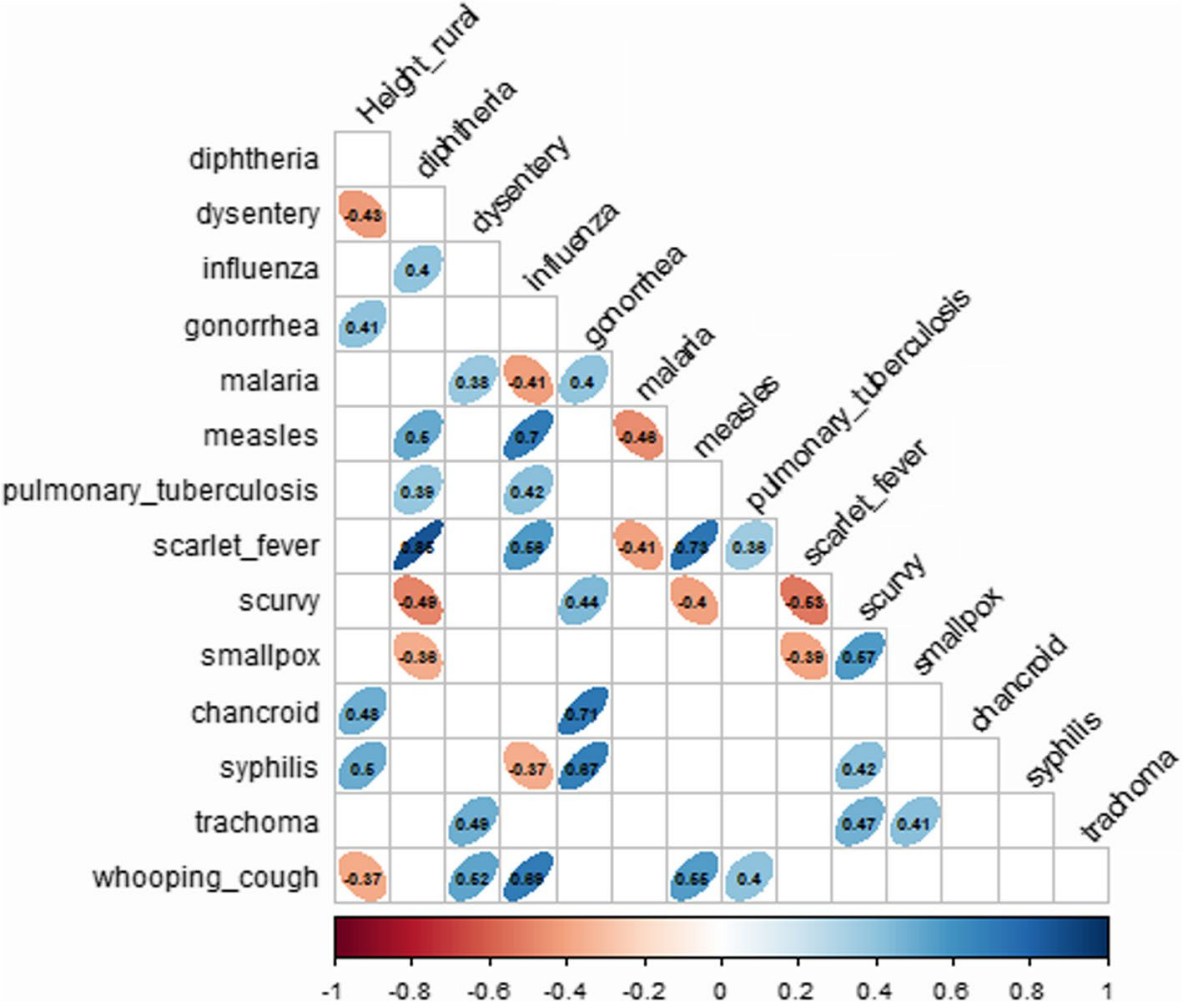
The correlation ratio: morbidity levels of certain illnesses vs. male body height in rural areas

Body height in urban areas was significantly but negatively correlated with the morbidity of dysentery (*r* = − 0.46, *p* value = 0.01), influenza (*r* = − 0.44, *p* value = 0.02), whooping cough (*r* = − 0.43, *p* value = 0.02), and trachoma (*r* = − 0.38, *p* value = 0.05). In addition, there was a positive correlation between the morbidity of the chancroid (*r* = 0.43, *p* value = 0.02) and the incidence of syphilis (*r* = 0.4, *p* value = 0.04).

Body height in rural areas was significantly but negatively correlated with dysentery morbidity (*r* = − 0.43, *p* value = 0.02) and whooping cough (*r* = − 0.37, *p* value = 0.02), as well as with venereal diseases such as chancroid disease (*r* = 0.48, *p* value = 0.01), syphilis (*r* = 0.5, *p* value = 0.01) and gonorrhea (*r* = 0.41, *p* value = 0.03).

It is necessary to explain the possible reasons for the different directions of the correlation ratio. It seems to be obvious when we speak about the negative correlation ratio—the greater the body height is, the lower the morbidity of particular illnesses. Thus, these illnesses are easily transmitted when living conditions are more unfavorable.

In what ways could we explain the positive correlation ratio? We should take a deep dive into the original dataset. We will see that the leaders of venereal diseases are the southern territories of the Soviet Union – Uzbekistan, Kazakhstan, Turkmenistan, and others. The illness was easily transmitted among the local ethnic population [18–27], but the values of body height used in the analyses and provided in the reference book [10] were about Russian conscripts, as Russian conscripts are usually greater than those of people of different ethnicities. According to the analyzed dataset, the difference could reach 2–3 cm [12].

**Fig. 3:**
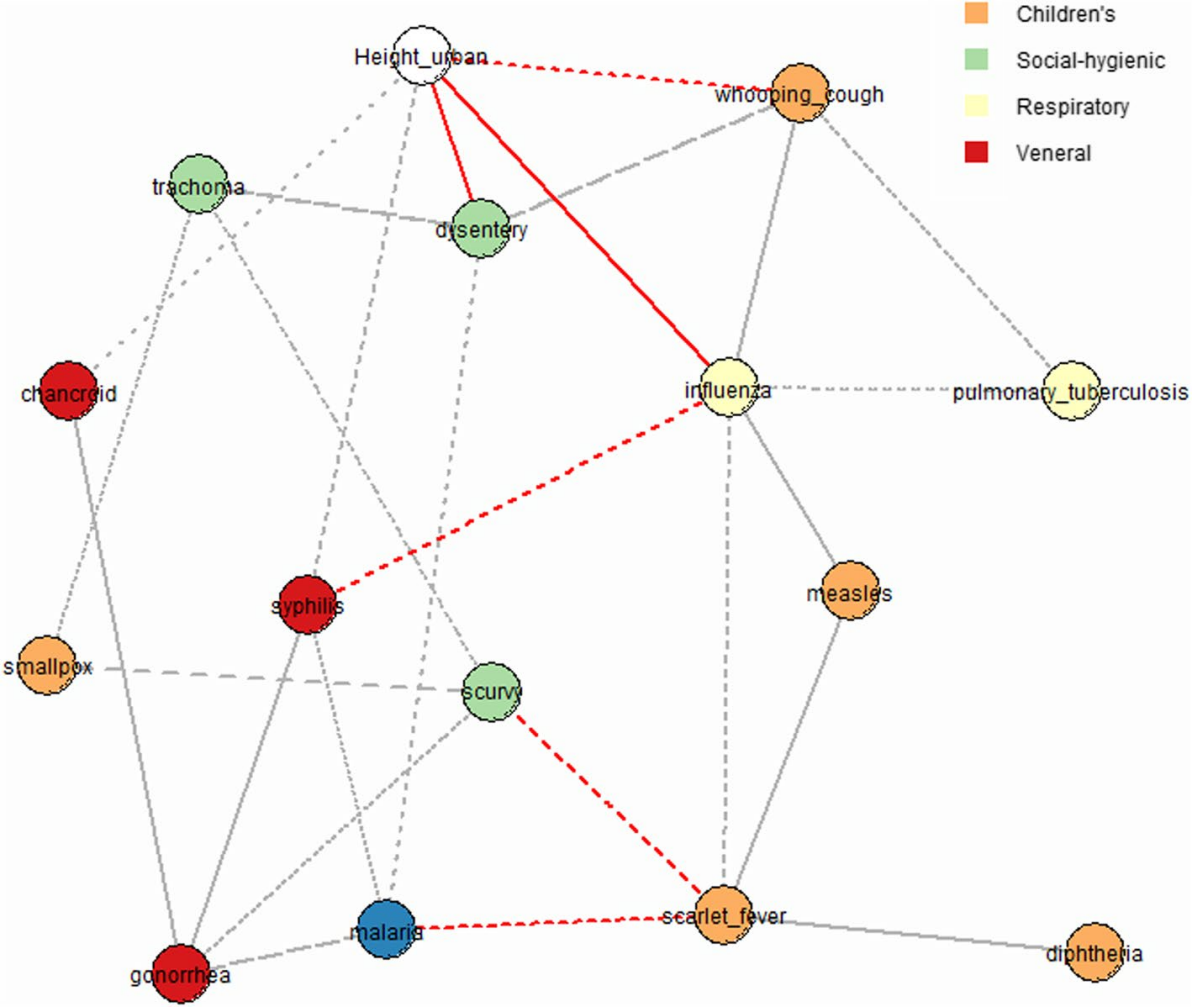
Associations between the values of body height in urban areas and the morbidity of various illnesses

Furthermore, SNHA graphs were constructed. Figure 3 shows that associations between the value of body height in urban areas and indicators of morbidity of influenza and dysentery were found in more than 75% of all dataset resamplings (solid lines); associations between body height values in urban areas and indicators of morbidity of whooping cough, syphilis, and trachoma were found in more than 50% of all resamplings (broken lines); and associations between body height values in urban areas and morbidity of chancroid were found only in more than 25% of all resamplings (dotted lines).

**Fig. 4:**
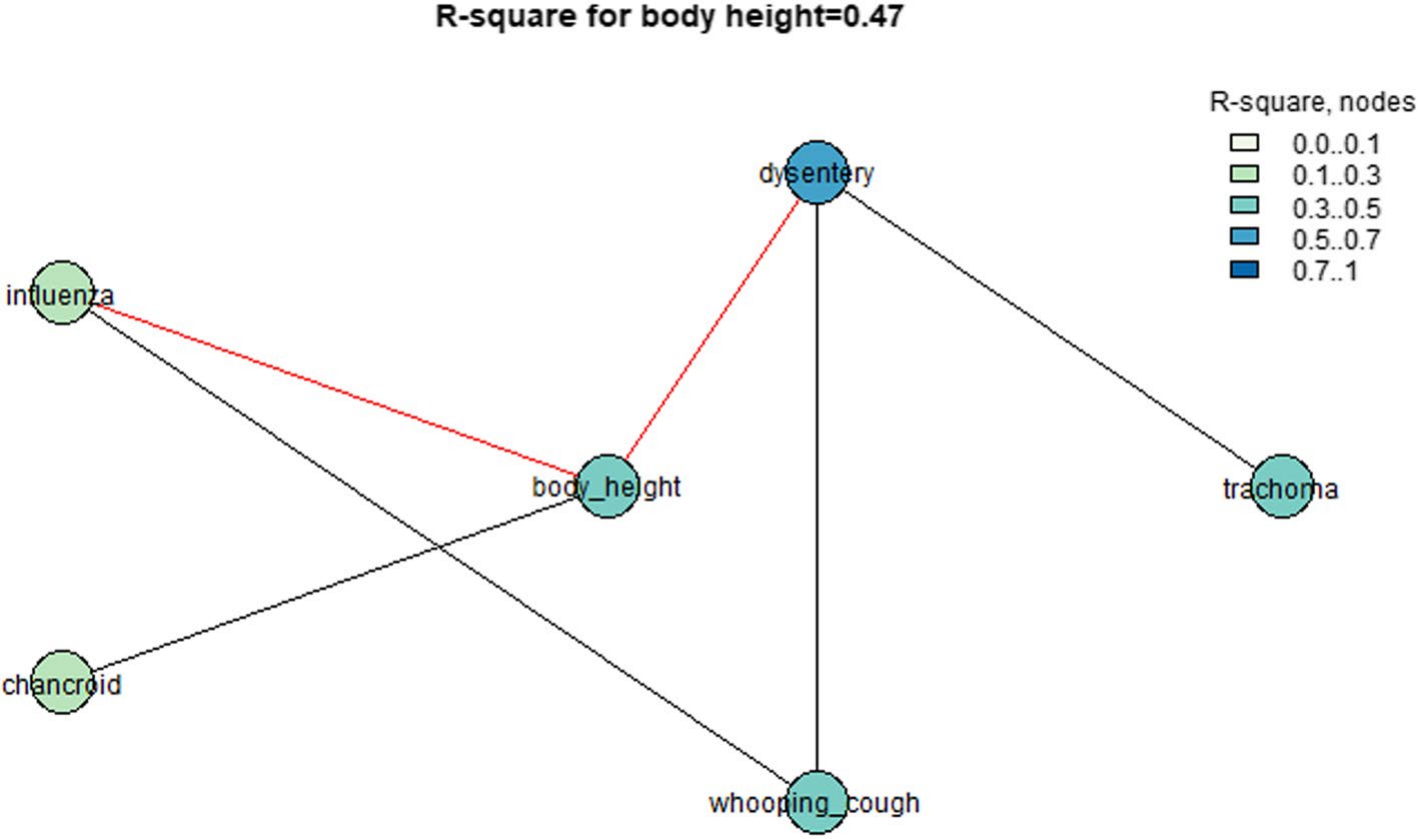
*R*-square values for the urban areas

Then, we calculated the R-squared values by a linear model for all nodes that were associated with the indicator of body height in urban areas. We used the function “SNHA_rsquare” [16]. The linear model predicts the node by an additive mode using its neighboring nodes in the graph. The mean R-square value for all variables associated with body height was 0.37, but the R-square value for body height was 0.47. Thus, the morbidity of diseases such as influenza, dysentery, and chancroid could explain up to 0.52 of the variation in body height in urban areas during the observed period (Fig. 4).

**Fig. 5:**
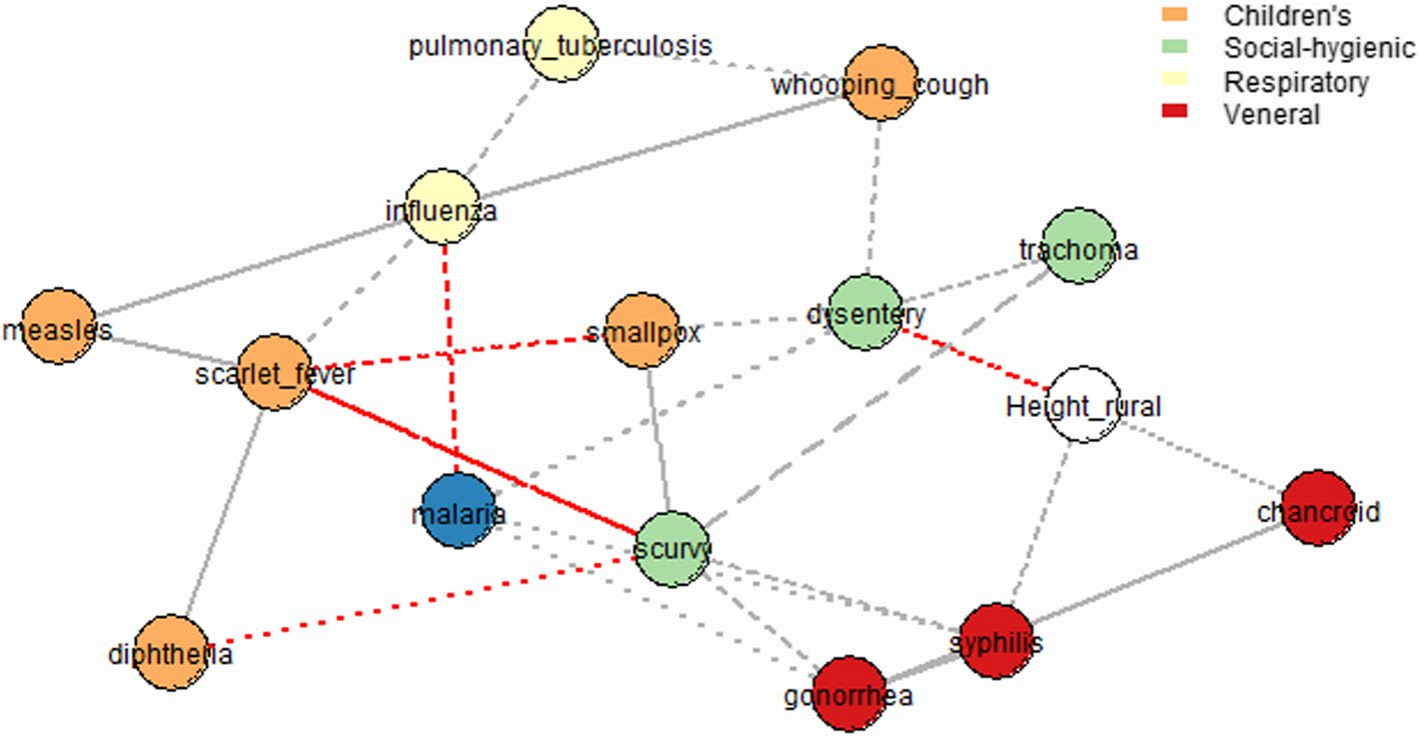
Associations between the values of body height in rural areas and morbidity due to various illnesses

**Fig. 6:**
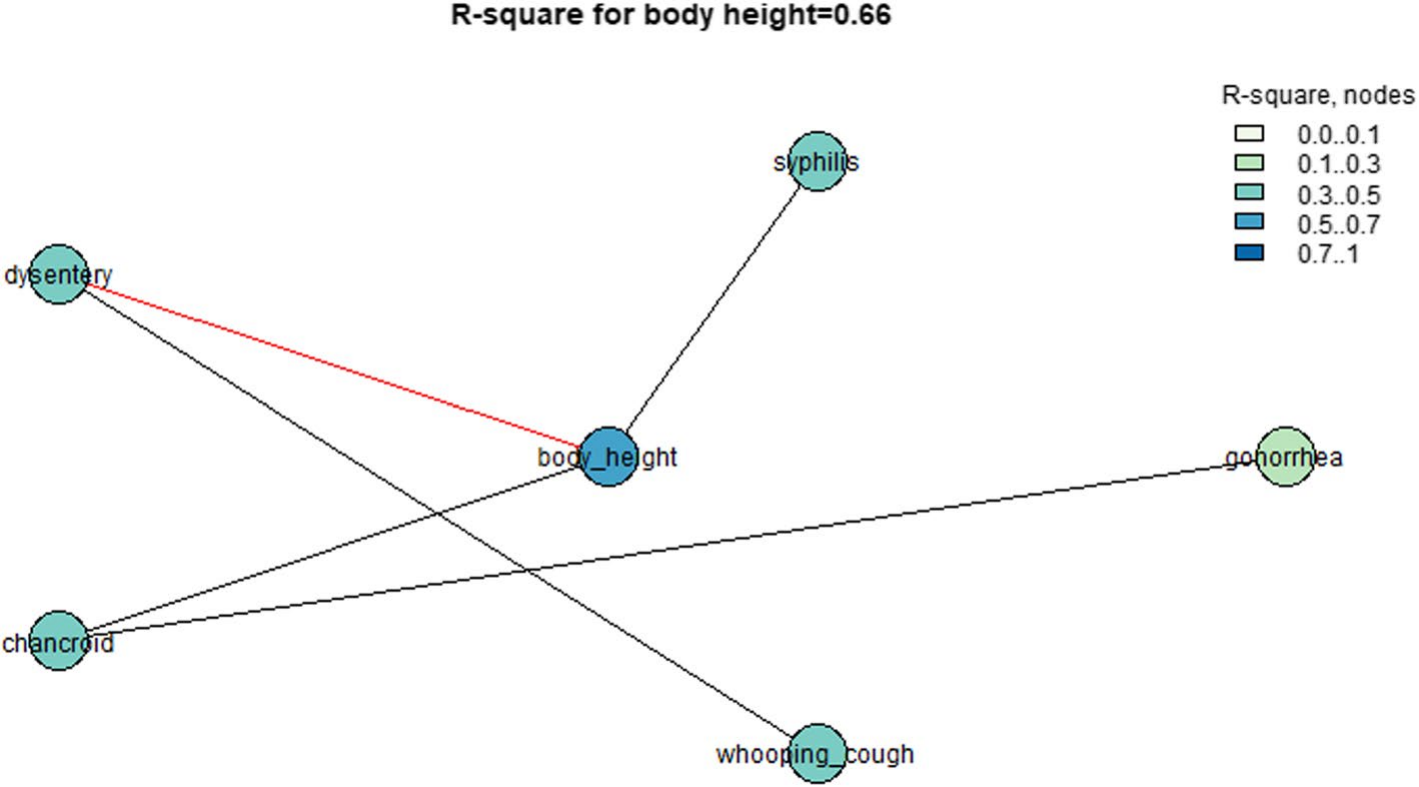
*R*-square values for the rural areas

The same steps were performed for the dataset for rural areas. Figures 5 and 6 show that body height in rural areas was associated mostly with the morbidity of venereal diseases (chancroid, syphilis) and was less strongly associated with gonorrhea and with the diseases transmitted due to low quality of life and low sanitation (dysentery and whooping cough). All of these associations could explain up to 0.43 variations in all the provided variables. Three variables (chancroid, syphilis, and dysentery) could explain up to 0.66 variations in body height in rural areas (see Fig. 6).

For this dataset, we also checked the associations between the morbidity of various illnesses and body mass index in the same territories; however, no significant correlations or associations were found for either urban or rural areas.

Finally, we investigated whether the childhood epidemiological situation of conscripts in the regions where they were growing up could influence their final body height and other physical parameters, such as body weight, BMI, and chest circumference.

**Fig. 7:**
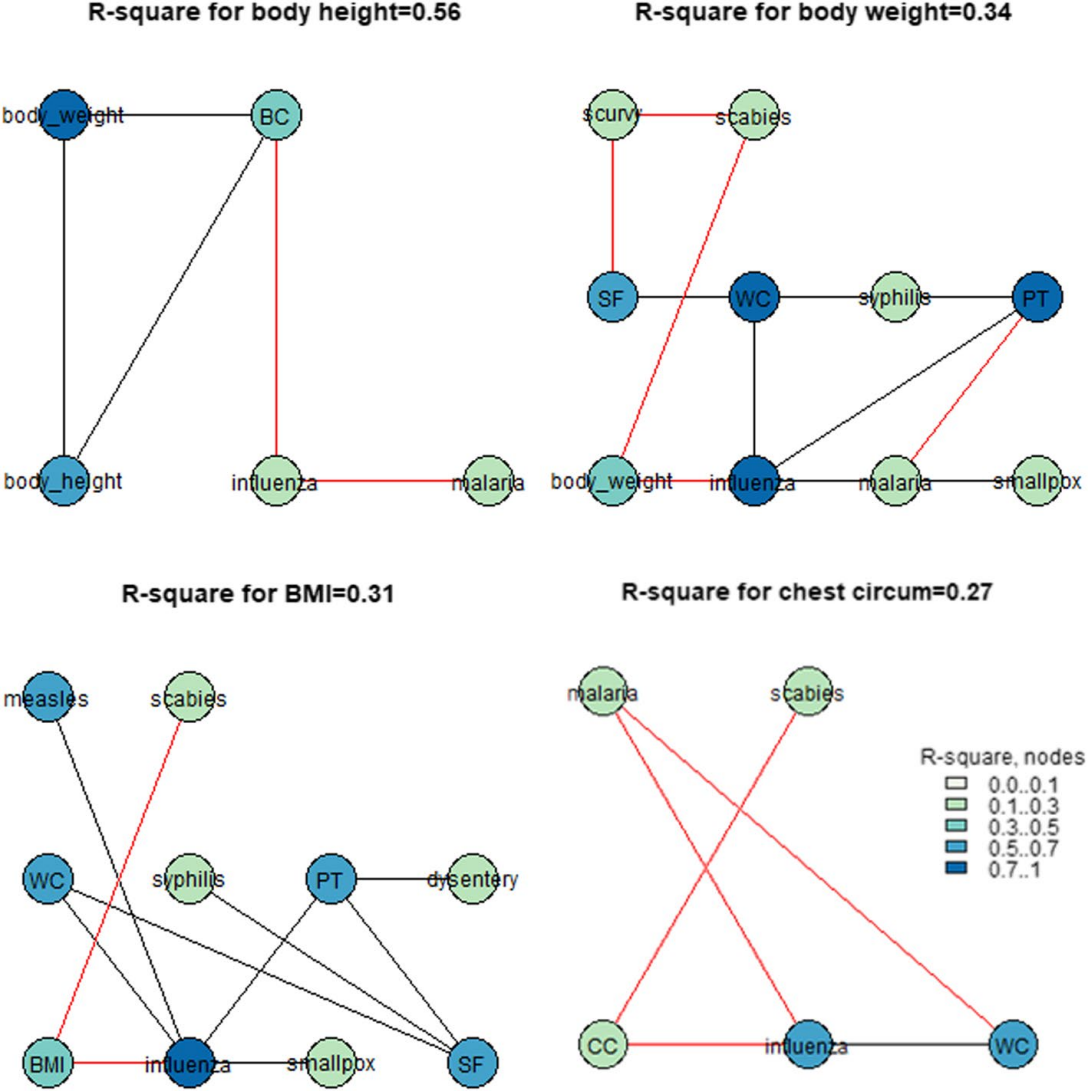
*R*-square values for the urban areas for the dataset that illustrates the level of morbidity during conscripts’ childhood. Abbreviations: CC—chest_circumference, PT—pulmonary_tuberculosis, WC—whooping_cough, SF—scarlet_fever

**Fig. 8:**
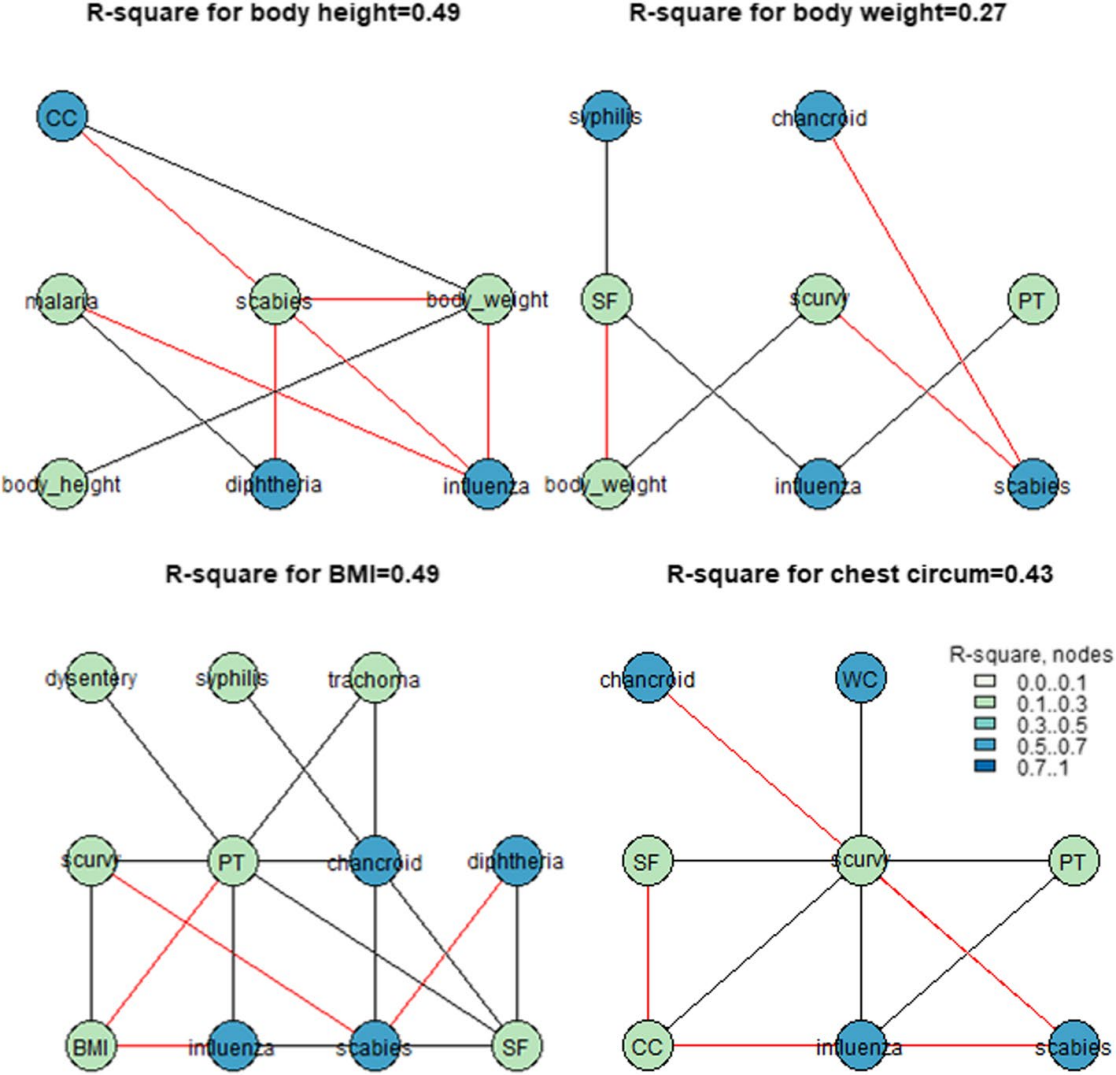
*R*-square values for the rural areas for the dataset that illustrates the level of morbidity during conscript childhood. CC—chest_circumference, PT—pulmonary_tuberculosis, WC—whooping_cough, SF—scarlet_fever

For every abovementioned trait, we followed the same steps as were previously described. Figures 7 and 8 illustrate the results of the calculation of four *R*-squared graphs for urban and rural territories. Thus, the values of conscripts’ body height could be up to 0.56 for the indicators strongly associated with body weight and chest circumference in urban areas and up to 0.49 for the indicators strongly associated with body weight in rural areas. No association between the level of morbidity in the regions during childhood and conscripts’ body height was found. Moreover, the conscripts’ body weight, chest circumstance, and BMI were significantly correlated with the morbidity of various illnesses, such as influenza, scabies, scurvy, scarlet fever, and malaria. These associations could explain up to 0.33 and 0.27 of the variation in conscripts’ final body weight in rural and urban areas, respectively. The final chest circumference of the conscripts reached 0.27 and 0.43, and the BMIs of the conscripts reached 0.31 and 0.49.

Therefore, we suggest that the morbidity of influenza, scabies, and other illnesses during childhood might be associated with the physical characteristics of the conscripts, such as BMI, weight, and chest circumference.

## Discussion

**Fig. 9:**
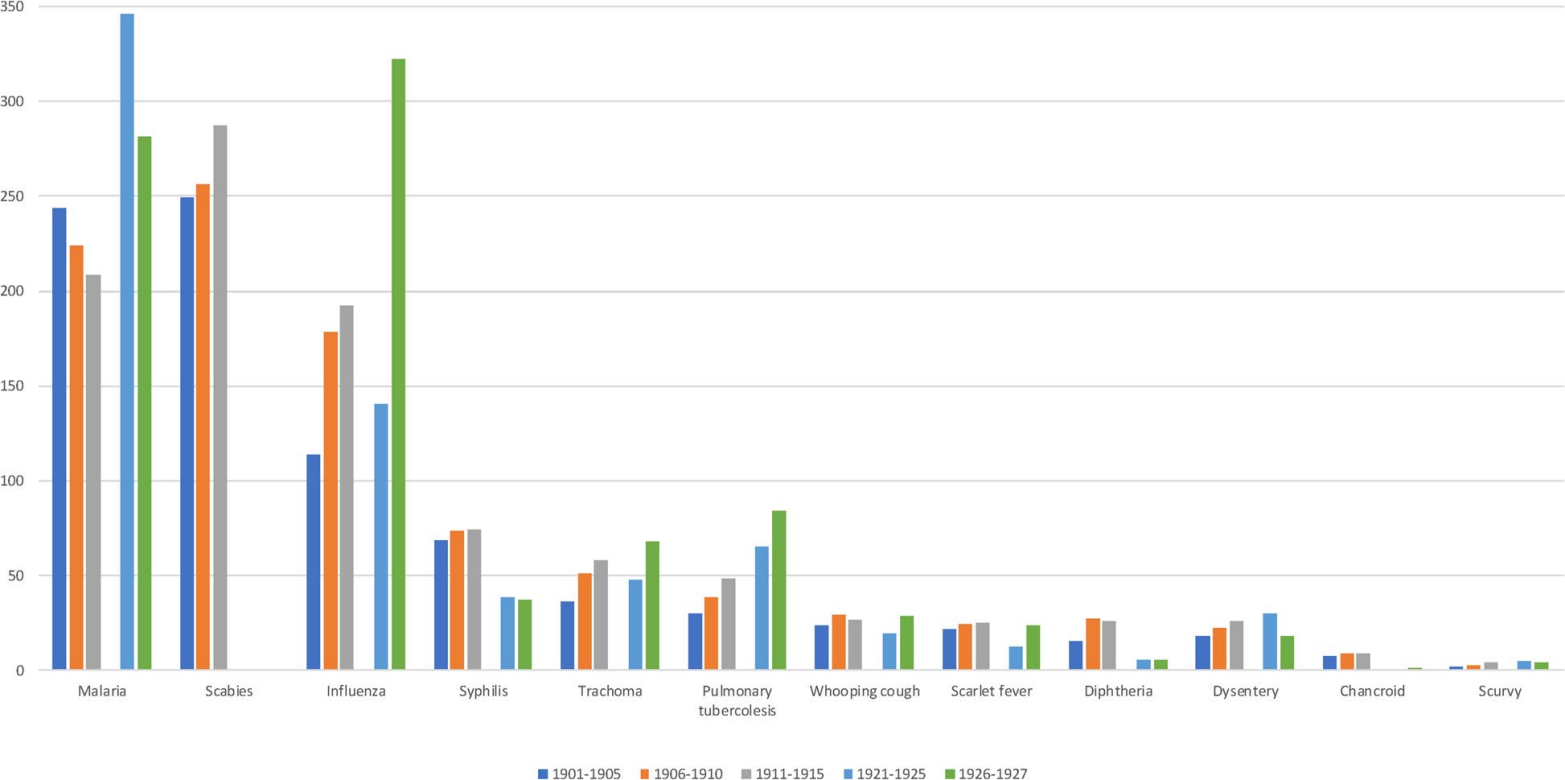
Levels of morbidity of different illnesses at the beginning of the twentieth century, per 10,000 people in Russia

The role of diseases has changed dramatically over the centuries. Some illnesses that we have considered in the analyses are currently forgotten or extremely rare in medical practice, such as malaria, scabies, or trachoma. Therefore, to understand what those illnesses indicated in the first decades of the twentieth century, we used descriptions from the Great Medical Encyclopedia by N. Semashko published in 1928–1936 in 35 volumes [18–27]. Every article about diseases contains the historical background of the disease, the statistical information on the prevalence of illnesses worldwide and in some particular territories, and its clinical description and epidemiology that was relevant to the period when the Encyclopedia was published. Moreover, to understand the prevalence of the diseases, we aggregated statistical information from the data sources used in the analyses [10, 13]. As shown in Fig. 9, there were three key diseases at the beginning of the century—malaria, scabies, and influenza. They are markers of unfavorable living conditions and a low level of sanitation culture.

*Malaria* is a mosquito-transferring infectious disease that affects humans and other animals. Children are especially prone to malaria. Malnutrition, migration, and low socioeconomic development of the territory could lead to severe epidemics, especially among immigrants arriving in malarial areas. The areas of high morbidity of malaria are traditionally located in the southern regions of the Russian Empire, namely, the Caucasus and South Asia, due to a lack of medical care and high population density. However, the morbidity of malaria was also high in the regions near the Volga River and Central Russia after the Civil War. The key reasons are as follows: a significant number of people migrated from the southern parts of the country to the North, and there was a sharp increase in the air temperature in 1921–1922, which led to the spread of wild soils and swamping due to the absence of normal agricultural activity. There was a dramatic decrease in in-livestock numbers and an overall decline in living conditions [18].

Another disease strongly associated with the lack of hygienic conditions was *scabies*. It is a contagious skin disease caused by the scabies mite. The level of morbidity of scabies increases during wars and among populations with low socioeconomic status and low sanitary conditions, with a lack or absence of health care. After the Civil War, the morbidity of scabies declined rapidly due to the development of the medical care system throughout the whole territory. Unfortunately, no statistical information was found to support this statement in our reference books or even in the Encyclopedia, except for one note about the level of morbidity in the city of Magnitagorsk in 1931. This figure was 27.6 per 10,000 people, approximately ten times less than that of the Russian Empire [19].

*Influenza* is a contagious disease that can cause both general symptoms (fever, headache, soreness in the muscles and back) and complications of organs in the upper respiratory tract. These complications usually become the causes of death. Influenza can spread pandemically. The most well-known influenza pandemic occurred in 1918–1919 just after the 1st World War and is known as the Spanish flu. Some influenza outbreaks were statistically recorded in 1926 and 1927 in Russia [20].

Before the First World War, the mortality rate from influenza in European countries averaged 10–20 people per 100,000 people. In 1918, this figure was 293 in Germany, − 336 in England, and − 471 in Sweden. At the same time, the mortality rate in these cities is two to three times greater [20].

During the period from 1908 to 1915, approximately 200 sick people per 10,000 people were registered annually in the Russian Empire. In 1927, there were 326 registered cases (in the USSR). Due to differences in administrative boundaries, it is problematic to compare the results for different territories. However, a comparison of the cities revealed the same trend as that in Europe—a significant increase in the number of cases in 1918–1919 and in 1926. Moreover, the morbidity in the Moscow region in 1906–1910 and 1926 was greater among children under 1 year of age and among adults aged 20 to 29 years, both among men and women. Usually, people of that age have more social contacts and a greater risk of being infected; thus, they can transmit illnesses more easily [20].

Other diseases that indicate low quality of life and lack of hygienic culture are trachoma, pulmonary tuberculosis, dysentery, and scurvy. According to our analyses, the incidence of these diseases during conscripts’ childhood was associated with BMI, body weight, and chest circumference. *Trachoma* is a chronic eye infection that leads to blindness. The spread of trachoma is related to the poor health status of the healthcare system, poor living conditions, and poor hygienic culture of the population (e.g., using common towels) [21]. *Scurvy* is a form of vitamin deficiency in which long-term vitamin C deficiency occurs. Scurvy occurs more frequently in malnourished individuals [22]. *Dysentery* is an infectious disease that spreads through household contact (through unwashed hands), water (through an infected water source), or food. Additionally, the role of flies as carriers of this disease is sometimes noted. The low socioeconomic status of the population and its lack of hygienic habits contribute to the spread of dysentery. The level of morbidity during the 1st WW in European countries and some cities of the Russian Empire supports this idea. Dysentery can also be associated with malnutrition and starvation. Due to these hardships, people become more susceptible to illnesses. Pulmonary tuberculosis is also an infectious disease that is associated with poor nutrition and low quality of life. People from the working class were exposed to this disease both in European countries and in the Russian Empire [23].

Other types of diseases associated with conscripts’ final height, such as syphilis and chancroid, are venereal diseases. Infection occurs mainly through sexual contact and less often through kisses, bites, and household items. These diseases spread due to the migration of people caused by unemployment or poor standards of living. Urban territories face these diseases more often than rural territories. The disease most often spreads among unmarried men aged 20–39 years with a low level of education and social position. They live in dormitories with low social and hygienic culture [24].

In rural territories of the Russian Empire and the USSR, syphilis was brought by soldiers and seasonal workers who were employed during the winter in these cities. This type was called social household syphilis and became widespread through the use of common household items. The low level of health education in the 1920s prevented the detection of disease in the early stages. In the USSR, the health care system started to organize a network of medical care services and to improve hygienic literacy. These methods help to decrease the level of morbidity and improve disease spread [24].

The last group of diseases included in the analyses were diphtheria, scarlet fever, and whooping cough. As shown in Fig. 9, the average morbidity was approximately 20–30 cases per 10,000 people. These diseases are usually spread between people by direct contact or through the air. They may also be spread by contaminated objects. Children who lived in houses with many family members and poor socioeconomic conditions were more likely to be infected. Thus, at the beginning of the twentieth century in St. Petersburg, the morbidity of scarlet fever was 18.5 cases per 10,000 people for wealthy people and 26.1 cases for poor people. Diphtheria most often affects children under 5 years of age, those with scarlet fever, and those with whooping cough under 9 years (Table 4). In the 1910s, there was an epidemic of diphtheria in Russia. The vaccine was invented only in the 1920s and helped to overcome this illness [25–27].

**Table 4:** The morbidity of scarlet fever and whooping cough among various age groups in Moscow city and the Moscow region in 1926 [26, 27]

	1926								1906–1908	
	Scarlet fever				Whooping cough					
	Moscow region		Moscow City		Moscow region		Moscow City		Moscow region	
	Male	Female	Male	Female	Male	Female	Male	Female	Male	Female
1 year and less	50	46	50	84	199	231	249	289	301.1	318.6
1–4 years	114	111	295	298	145	177	317	360	249	297.1
5–9 years	77	88	237	255	64	76	140	145	115.4	142.4
10–14 years	27	35	78	89	10	13	12	18	21.3	29.4
15–19 years	9	9	24	23	1	1	1	1	1.7	3.8
20–29 years	4	3	7	9	**1**	3	**1**	1	0.6	2

Thus, our hypothesis regarding the influence of the epidemiological situation in childhood on final body height and other physical parameters is as follows: body weight, BMI, and chest circumference were partially confirmed. Moreover, body weight, BMI, and chest circumference was associated with the prevalence of diseases spread among children.

## Conclusion

Our analyses revealed direct associations between the morbidity of some diseases and male body height and other anthropometric parameters. Our results revealed the morbidity of such illnesses as influenza, dysentery and chancroid could explain up to 0.52 of the variation in body height in adults in urban areas. Three variables (chancroid, syphilis and dysentery) could explain up to 0.67 variations in body height in rural areas. There are no associations between conscripts’ final body height and the level of morbidity during childhood. However, other final morphological characteristics such as BMI, weight and chest circumference could be associated with the level of morbidity of malaria, scabies, scurvy and scarlet fever during conscripts’ childhoods in urban and rural areas. The prevalence of these diseases could be strongly connected with poor living conditions.

## Limitations

Our analysis has several limitations. The first limitation is connected with the absence of statistical information on final body height for women. Thus, we could not confirm our conclusions for a significant part of the population. The statistical data about the morbidity rate for the whole population were used. The second limitation concerns the period of our analyses. The collapse of the Russian Empire led to significant changes in the administrative system, government boundaries and the system of collecting vital statistics. All these factors greatly influence the ability to organize the data for analysis. However, we extracted and considered as much information as possible from the statistical reports and other resources.

## Data Availability

The datasets used and/or analyzed during the current study are available from the corresponding author upon reasonable request.
